# Multifaced Generation of MOF Coatings via Vapor-Phase Sublimation and Deposition Reactions

**DOI:** 10.1021/acsami.5c16493

**Published:** 2025-12-15

**Authors:** Shu-Man Hu, Chin-Yun Lee, Yu-Ming Chang, Fang-Yu Chou, Hui-Hsuan Wang, Yu-Chih Chiang, Chen-Chi Wu, Hsien-Yeh Chen

**Affiliations:** † Department of Chemical Engineering, 33561 National Taiwan University, Taipei 10617, Taiwan; ‡ Department of Chemical Engineering, National Taiwan University of Science and Technology, Taipei 10607, Taiwan; § School of Dentistry, Graduate Institute of Clinical Dentistry, 33561 National Taiwan University, Taipei 10048, Taiwan; ∥ Department of Otolaryngology, 38006 National Taiwan University Hospital, Taipei 10018, Taiwan; ⊥ Molecular Imaging Center, 33561 National Taiwan University, Taipei 10617, Taiwan; ∇ School of Dentistry, Kaohsiung Medical University, Kaohsiung, 80708, Taiwan

**Keywords:** metal−organic framework, coating, vapor sublimation, multifunctionality, chemical vapor deposition

## Abstract

A general fabrication method for metal–organic framework (MOF) coatings through a vapor sublimation and deposition process is reported. The vapor-phase fabrication mechanism relies on the interplay between thermodynamic properties and the solid–vapor interface reaction. The processing parameters of ion concentration, vapor pressure, and system temperature enable control of the sublimation rate, and ion–linker nucleation and coordination reactions occur at the dynamic solid–vapor interface to form the proposed MOF coatings. The reaction rate is controllable and is proportional to the sublimation rate during the fabrication process. On the basis of the proposed fabrication mechanism, experiments are facilitated to obtain combinations of multiple metal cores and a variety of functionalized linkers for vapor deposition from solid solution sublimation. The fabrication process involves pore size control, crystalline orientation customization, and encapsulation of cargo to form composite coatings, and these processes are implemented during the same one coating fabrication process. The initiation and manipulation of the nucleation radius and thus the nucleation rate are easy to control and are related to the fabricated MOF coatings with tunable crystalline morphologies. Moreover, the MOF coating thickness is a time-dependent process that is proportional to the sublimation rate and deposition time. In the present study, the surface area of the fabricated MOF coatings was 1250 m^2^/g, the Young’s modulus was 2.8 GPa, the surface roughness was 238.7 ± 17.3 nm, and thicknesses ranging from 200 nm to 5 μm were prepared. Maximized compatibility is also discussed through the exploitation of solid solutions that are free of organic solvents and a purely dry process, and the final MOF coatings are conformal with high fidelity regardless of the substrate material type and the complexity of the geometries in both 2D and 3D. The stability of the MOF coating is inspected by an adhesion test that reveals the highest adhesion standard in the ASTM D3359 classification. The cell culture on this coating verified high cell viability with promoted cell attachment, proliferation, and increased osteointegration activities.

## Graphical abstract

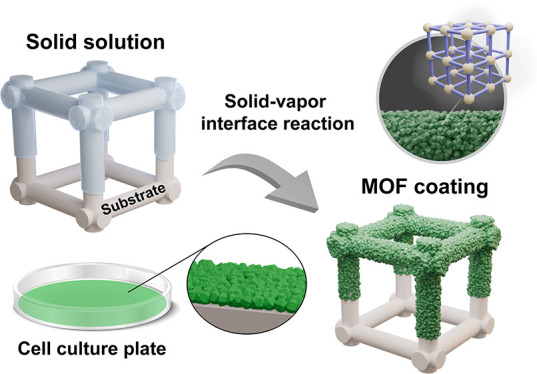

## Introduction

Conventional coating technologies are essential for improving the surface properties of materials with controlled hydrophobicity,^1^ improved mechanical durability,^2,3^ corrosion and/or fouling resistance,^4^ and increased biocompatibility.^5,6^ The development of more advanced coatings, in addition, offers control over the physical/structural properties of coatings with compartmentalized, layered configurations of coating composites and/or chemical properties, such as multifunctional chemical conduct for enabling multiple tasks,^7^ controlled gradients of compositions and chemical reactions,^8,9^ renewable/self-healing,^10^ switchable,^11^ and biodegradable activities,^12^ and/or combinations of the above physical and chemical variations,^13^ for the coated surface and devices. The coating technologies are produced in either solution-based or vapor-deposited fashions. The more traditional solution-based methods offer cost-effective and mass production advantages;^14^ however, their vapor-based counterparts have more striking merits, such as being a drying process to avoid moisture and solvents,^15^ a precisely controlled coating thickness,^16^ and conformal coverage even for complex geometries of the surfaces and/or devices.^17^

Metal organic frameworks (MOFs), a class of porous crystalline materials composed of metal ions coordinated to organic linkers, are mostly produced by solution-based approaches^18^ because of their high porosity and surface area and adaptable chemical properties. These solution-based methods, such as solvothermal synthesis, microwave-assisted,^19^ sonochemical,^20^ electrochemical,^21^ and microfluidic approaches,^22^ are prepared with postcasting,^23^ spin coating,^24^ spray coating,^25^ etc.,^26,27^ to form thin-film coatings on substrates. Recent efforts have also explored vapor-based strategies for MOF fabrication, such as molecular layer deposition,^28^ generalized vapor–phase linker exchange,^29^ and metal–organic chemical vapor deposition technology;^30,31^ applications using MOF coatings across multiple fields in materials science have shown promise.^32,33^ With the urgent need to fulfill the requirements for advancing and sophisticating new materials, the development of prospective MOF coatings is envisioned to be fabricated with a general and easy process involving simple steps, new processing mechanisms with the ability to control physical properties and chemical functionalities, avoidance of the use of solvents to sustain biocompatibility and lower environmental impacts, and important compatibility with complex topologies and geometries on substrates and devices from 2D to 3D. Compared with conventional vapor-phase techniques such as molecular layer deposition, which are often limited by slow surface reaction kinetics, complex vacuum control, and substrate dependence, the present sublimation-assisted vapor deposition offers a simpler, solvent-free route under mild thermodynamic conditions. This dynamic solid–vapor interface enables rapid coordination reactions and uniform coating formation without the need for preactivation or seed layers. Moreover, the process can be readily extended to diverse substrates and compositions, highlighting its scalability and adaptability. In this study, we report a straightforward and general process that uses solid-phase metal solutions as templates for the vapor deposition of organic linkers and the formation of controllable and functional MOF thin coating layers on substrate surfaces. More specifically, the process exploits a dynamic solid–vapor interphase reaction where a nonreactive solvent sublimates and sterically stabilizes the reactant of an organic linker in the solid phase prior to the reaction; subsequently, the solid reactant is subjected to a change in thermodynamic conditions at a pressure down to approximately 200 mTorr and at room temperature or above 0 °C. Under such thermodynamic conditions, the sublimation process separates the system molecules from the solid solution, i.e., water molecules are sublimated from the solid phase to the vapor phase, and mass transport of vapor escaping the solid system occurs. In contrast, the solute molecules of the metal ions favored their original and stationary state with no mass transport under the same conditions due to rejection from the pure iced crystal during the propagation of the water/ice crystallization process. These functional elements exhibit steric stabilization within the freezing interface, the directed and/or random motions of the molecules are suppressed,^34^ and mass transport greater than the molecular scale does not occur even with an unsteady state mass transfer configuration.^35^ Through the sublimation of water molecules and the deposition of organic linkers at the solid–vapor interface, a metal–linker coordination reaction is facilitated, resulting in the growth of MOF thin films on the substrate surfaces. Chemical vapor deposition (CVD) has been proven to be a drying process with unrivaled coating conformality toward surface roughness and device geometry and is particularly useful for moisture-sensitive, miniaturized, nonflat, delicate surfaces and/or devices. This approach enables uniform growth of MOF coatings on solid substrates under controlled conditions without the need for extensive postsynthetic modifications while preserving the structural integrity and functionality of the MOFs.

The proposed vapor-phase fabrication method of MOF coatings has several advantages, including (i) being a dried vapor-phase process that avoids solvation and hydration processing and does not compromise undesirable complex structural reorganization transitions; (ii) being able to control the chemical composition and physical properties on the basis of the stagewise solid–vapor reaction and sublimation rate; (iii) having facile adjustability over the sublimation rate of the solid template, which enables control of the resultant MOF morphologies; (iv) being able to access theoretically secondary, ternary, quaternary, and other reaction pathways to produce functional and hybrid MOF coatings with configured structures and/or distinct compartment boundaries; (v) having uniform MOF coatings on substrates regardless of the material type and geometric complexity; and (vi) having a wide range of functional MOF coatings that are fabricated in one fabrication process. This approach represents a rapid and robust fabrication route of functional materials to metal–organic framework coatings on surfaces and devices.

## Results and Discussion

A vapor-phase reaction pathway is proposed, wherein the vapor acts as the molecular precursor for organic linkers, whereas a solid-state solution functions as the framework harboring metal ions. The organic linkers are delivered via vapor-phase diffusion to the solid template, initiating a solid–vapor interface reaction under low-pressure conditions. As the solid solution undergoes sublimation, the underlying, nonvolatile metal ions become progressively exposed on the surface, enabling coordination reactions between the metal ions and the vapor-phase organic linkers. This interaction at the solid–vapor interface drives nucleation and subsequent crystallization into a MOF framework. As shown in Figure 1a, the solid-state precursor solution allows for the selection of various metal ions, while a variety of organic linkers can be introduced in the vapor phase, enabling the synthesis of MOF coatings with tunable properties. During this process, the localized vapor pressure and temperature gradient near the solid–vapor interface provides the thermodynamic driving force for nucleation. As the coordination reaction proceeds, metal–linker complexes gradually assemble into ordered frameworks, accompanied by surface reconstruction and crystal growth. The dynamic balance between sublimation rate and linker diffusion determines the thickness and uniformity of the resulting MOF layer. Such a mechanism is consistent with previous reports on vapor-phase MOF growth under low-temperature conditions, supporting the proposed formation pathway.^36,37^ As the reaction progresses, the sublimation of water molecules from the solid solution (i.e., sublimation of ice) follows a characteristic zero-order kinetic behavior,^38,39^ in which the rate of sublimation remains constant over time at a fixed temperature and pressure; with the continuous occurrence of sublimation, the nonvolatile metal ions within the solid solution are progressively exposed to the outer surface, where a dynamic solid/vapor interface emerges in three dimensions at any encountered coordinate, and the exposed ions and deposited organic linkers engaging with the coordination reaction occur on this dynamic interface to obtain the accordant metal organic frameworks. The concentration of the metal ions is given by the mass transport of sublimation and follows Fick’s law,^40,41^ that its directional concentration gradient d*C*/d*x* is linearly proportional to the sublimation rate in the corresponding direction d*x*. The overall nucleation and growth of MOF coatings on the surface, according to classical nucleation theory,^42,43^ is also proportional to the concentration gradient and thus to the same sublimation rate in all directions. The kinetic behavior of ice sublimation and its impact on the metal ion concentration can be explained through ice sublimation dynamics, vapor-phase linker adsorption, metal ion diffusion, and nucleation theory. The variation in the metal ion concentration affects the nucleation rate, thereby affecting the final pore structure characteristics of the MOF. The theoretical calculations and equation deductions for the interplay between sublimation thermodynamics and the interface nucleation kinetics of the MOFs are included in the Supporting Information, eqs S1–S3. During the experiments, a zinc solid solution was used for the sublimation and the vapor deposition of organic linkers of 2-methylimidazole to result in the proposed fabrication of zinc-MOF coatings on a silicon substrate, and as further evidenced by the cross-sectional morphology shown in Figure 1b. A mass spectrometric residual gas analyzer was utilized for the detection and monitoring of the vapor-phase molecular systems during the fabrication process. The results of the molecular mass data in Figure 1c show strong peaks at 18 and 82 amu, indicating the presence of water molecules (sublimation) and 2-methylimidazoles (deposition) during the reaction. Additional vapor deposition of linkers, including terephthalic acid and imidazole, was also attempted with, and the results are included in the Supporting Information in Figure S1. The real-time monitoring in Figure 1d also revealed a gradual reduction in water signals (normalizing to background signals of nitrogen N_2_), further indicating the depletion of sublimated water vapor, with an eventual slope approaching zero, which can indicate the completion of the fabrication process. The linear trend of decreasing water signals revealed a constant sublimation rate, and the collective XRD data of the confirmed MOF signals in Figure 1e also showed consistent linear dependency for the same time, unambiguously supporting the suggested fabrication mechanism. Furthermore, with proper stoichiometric ratios to feed the above-mentioned solid–vapor interface reaction mechanism of the proposed MOF coating fabrication process, devised MOF coating products were successfully produced on substrate surfaces. As revealed in Figure 1f,g, a uniform coating with detailed highly crystalline network structures was observed for the tested zinc and cobalt solutions with linkers. Detailed structural analysis by scanning electron microscopy (SEM) and transmission electron microscopy (TEM) confirmed an anticipated rhombic dodecahedron structure that was comparable to the well-known zinc–imidazole zeolitic imidazolate framework.^44^ Unambiguously, similar highly crystalline network structures were also discovered on a cobalt-MOF coating, and high-contrast images were recorded via a polarized microscope. Atomic force microscopy (AFM) further revealed the high crystallinity of the fabricated cobalt-MOF coatings, yet a uniform surface morphology was discovered with a root-mean-square roughness (*R*_RMS_) of approximately 238.7 ± 17.3 nm. The mechanical properties of the MOF coatings were also measured, revealing a surface area of 1250 m^2^/g, a Young’s modulus of 2.8 GPa, and thicknesses ranging from 200 nm to 5 μm. The corresponding stress–strain curve from dynamic mechanical testing, which further confirms the mechanical stability of the coatings, is provided in the Supporting Information in Figure S2. Interestingly, if the stoichiometric ratios of the used starting materials were suboptimal in the vapor-phase nucleation process, the fabrication may result in disordered and metastable coating products due to incomplete or improper coordination between the metal ions and the organic linkers; observations included coatings with organic linker precipitates, partially reacted metal oxides, and amorphous molecular structures lacking long-range ordered crystalline lattice characteristics of the MOFs. These suboptimal results are also included in the Supporting Information in Figures S3.

**Figure 1:**
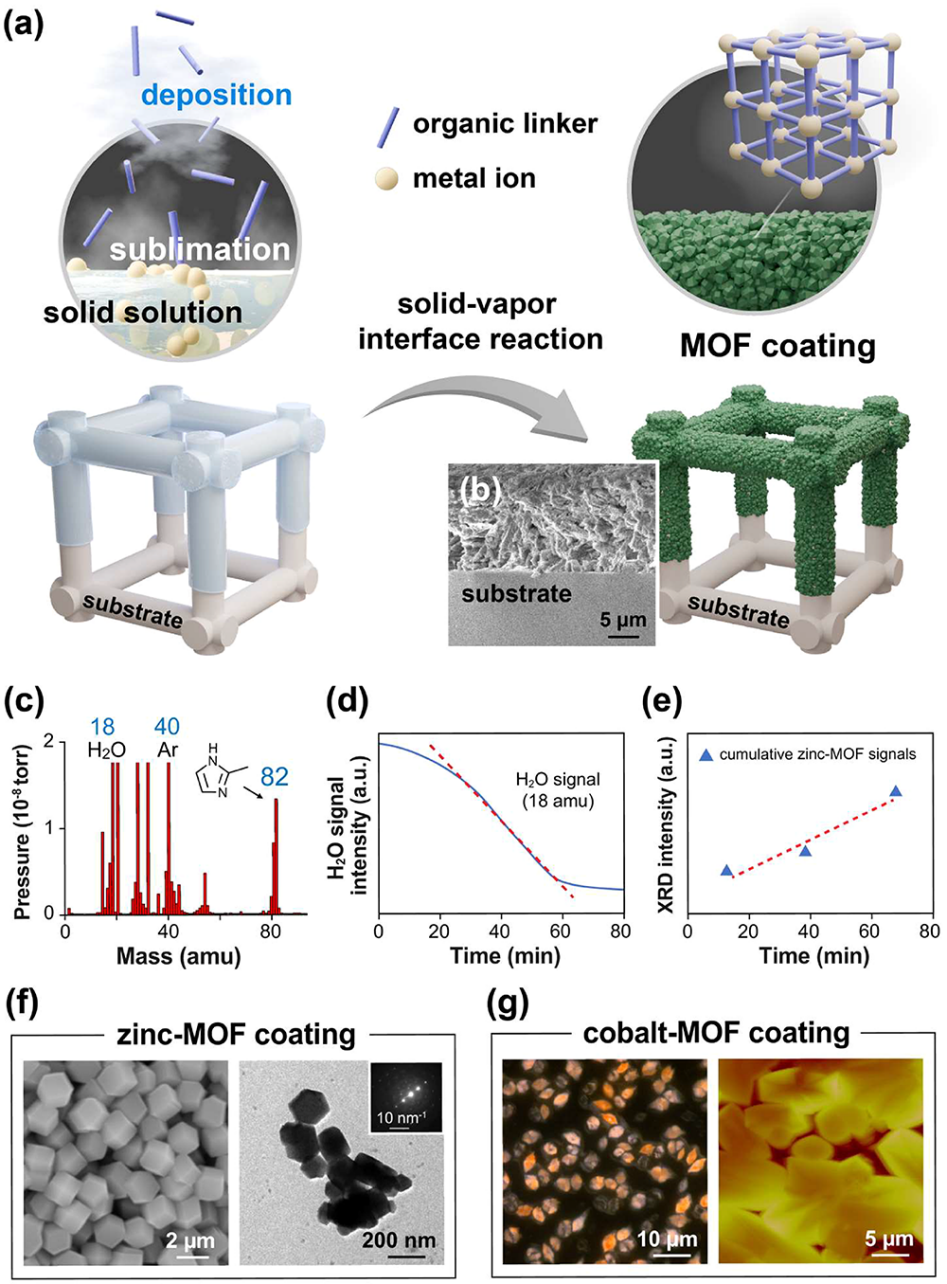
MOF coatings fabricated by vapor-phase reactions and processes from solid solutions. (a) Schematic of the proposed MOF coating process via vapor sublimation and deposition, where the organic linkers in the vapor phase are deposited on a solid solution containing metal ions. (b) SEM image of the cross-section of the fabricated MOF coating on a glass substrate surface. (c) Vapor-phase organic linker 2-methylimidazole was detected by an in situ mass spectrometry gas analyzer at 82 amu during the fabrication process. (d) Real-time plot of the vapor-phase signals of water molecules and (e) time plot of the XRD signals at 15.1, 27.0, and 30.5° from the fabricated zinc-MOF coatings. The relative correlation between (d) and (e) the cumulative MOF formation signal and reaction time. (f) SEM and TEM images showing the consistent and uniform rhombic dodecahedral morphology of the fabricated zinc-MOF coatings. The inset image also confirmed the diffraction pattern. (g) Microscopy images obtained by polarized microscopy and AFM revealed similar highly crystalline and uniform results for the fabricated cobalt-MOF coatings.

The ubiquitous desire to introduce functionalities into MOFs during synthesis with mixed linkers,^45^ and/or through postsynthetic modifications,^46^ improved their internal and interfacial properties,^47^ e.g., increased selectivity in gas separation and storage uptake,^48^ changing framework channel size, flexibility, hydrophobicity,^49^ and improved efficiencies in catalytic and enzymatic systems.^50^ Cyclophane-based molecular systems have proven chemical with well-developed functionalization abilities for aromatic mono-, di-, and tetra-substituents,^51,52^ delivering multifunctional capabilities, and processing facilities in vapor-phase (macro)­molecule conjugation^53^ and polymerization systems with a variety of functionalities for precise molecular-level control,^54^ making them ideal for fabricating the proposed vapor deposition of MOF coatings herein. In this study, cyclophanes with aminomethyl and trifluoroacetyl functionalities were exploited for their proven vapor precursor properties, transformed in the same vapor phase into functionalized terephthalic acid linkers and deposited on a zinc-based solid solution with the proposed fabrication mechanism to produce functionalized MOF coatings. Notably, the vapor-phased controls over multiple cyclophanes/terephthalic acids with stoichiometric ratios were rather flexible to adjust during vapor deposition and shared the same transport behavior in both the process and the solid solution. A schematic of the fabrication of this vapor-phased, multifunctional MOF coating is shown in Figure 2a, and theoretically, functionalization with two or more functional conducts is multiplied in the proposed mechanism, designer recipe ratio controls and flexibility; is accessible in the same and one single process; and more complicated solid–vapor interfaces can be managed, i.e., (1) multiplied and ratioed linkers to coordinate with one type of exposed ion on the same solid solution, (2) multiple linkers with more than two types of ions with administered concentrations in the solid solutions, and combinations of (1) and (2). The ability to functionalize and fabricate MOF coatings comprising tic-tac-toed metal cores and fractal functionalities in the final framework structure in a vapor fabrication process is a state-of-the-art method of MOF coating generation. At the time of this study, no general and functional MOF fabrication process has been reported without the requirements of multiple and complex modification steps, and conventional and solution-based processes cannot produce such functional MOF coatings in simple steps. Characterization by an in situ residual gas analyzer confirmed the proposed vapor-phased functional linkers of terephthalic acid, and as indicated in Figure 2b, the mass peaks at 131, 151, 174, and 200 amu revealed the presence of the accordant terephthalic acid. Further verification of the aminomethyl and trifluoroacetyl functionalities on the fabricated MOF coatings by X-ray photoelectron spectroscopy (XPS) analysis, as indicated in Figure 2c, revealed that the recorded fluorine (F) and nitrogen (N) signals from the aminomethyl and trifluoroacetyl groups corresponded to the anticipated elemental concentrations and combinations in the fabricated MOF coatings and that 1:1, 3:1, and 5:1 ratios were confirmed for these multifunctional MOF coatings. Moreover, evaluations of the specific reactivities of aminomethyl and trifluoroacetyl on the fabricated MOF coatings were performed by reacting with fluorescence-labeled *N*-hydroxysuccinimide (NHS) ester in the blue channel and hydrazide in the red channel. Localized immobilizations in square array patterns were performed for these probing molecules to guide vision, and the fluorescence signals were detected by both a fluorescence microscope and a confocal laser scanning microscope. The results are shown in Figure 2d,e. Characterizations and comparisons of the fluorescence intensities were also consistent for the deployed functionality ratios of 1:1 and 3:1, as shown in Figure 2f. Additional verification of the functionalities and ratios of the MOF coatings by using energy-dispersive X-ray spectroscopy is included in the Supporting Information in Figure S4.

**Figure 2:**
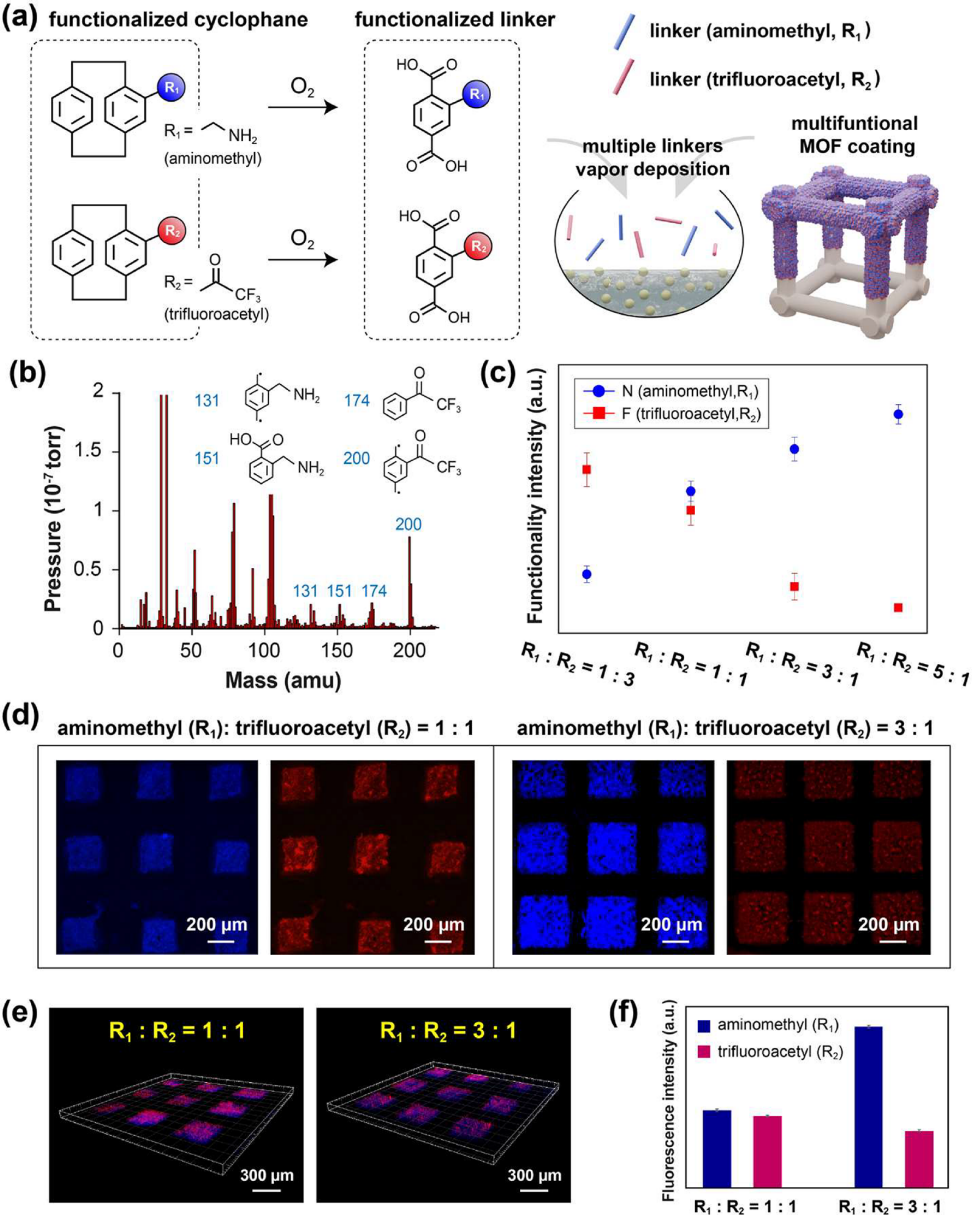
Controlling functionalization in the vapor phase of linkers. (a) Schematic of the use of vaporized and functionalized cyclophanes and their conversion to the corresponding terephthalic acid linkers for vapor deposition to produce multifunctional MOF coatings. Selected functionalities of aminomethyl (R_1_) and trifluoroacetyl (R_2_) were used in the study. (b) Detection of the functional terephthalic acid linker in (a) by a mass spectrometric gas analyzer. The characteristic peaks at 131 and 151 amu corresponded to the aminomethyl groups, and the peaks at 174 and 200 amu were attributed to the trifluoroacetyl groups. (c) XPS analysis showing the relative elemental concentrations of nitrogen and fluorine with deposition ratios of 1:1, 1:3, and 1:5 used to fabricate multifunctional MOF coatings. (d) Images recorded by fluorescence microscopy and comparisons of fluorescence signal intensities for different ratios of functional groups. Specific labeling reactions involving the use of NHS ester in the blue channel and hydrazide in the red channel confirmed the administered ratios of 1:1 and 3:1 for aminomethyl and trifluoroacetyl, respectively, on the coatings. (e) Confocal laser scanning microscopy images showing a uniform distribution of the fluorescence signals over a 0.75 μm in the *z*-axis corresponding to the thickness of the MOF coatings fabricated. (f) Second and averaged fluorescence intensity analysis unambiguously confirmed the 1:1 and 3:1 ratios.

Furthermore, the ability to maximize porosity and control structural coordination as well as the crystalline domain was achieved during the vapor-phase process of fabricating the proposed MOF coatings. On the basis of the aforementioned mechanism for the vapor process, the sublimation rate is parallel to the reaction rate, and theoretical control of the thermodynamic properties can dictate the solid–vapor interface reaction kinetics. The theoretical calculations and equation deductions for the interplay between sublimation thermodynamics and the interface nucleation kinetics of the MOFs are included in the Supporting Information, eqs S4–S6. We thus hypothesize that controlling the thermodynamic processing parameters, including the ion concentration, temperature, and pressure, can result in the customization, during the fabrication reaction, of the crystallographic orientations and the framework structure of the final MOF coating products. For example, while the ion concentration and pressure are held constant, the varying temperature directly dictates the sublimation rate and thermodynamic equilibrium state; the nucleation and molecular collision-driven MOF formation reactions involve thermodynamic-controlled or kinetic-controlled reactions, and the timing and speeding rate of vapor sublimation affect the framework/crystalline formation patterns. In the experiment, accelerated sublimation favored kinetic control and remained in a nonequilibrium thermodynamic state, which can result in MOF structures with metastable morphologies; distinct and unpredictable results of crystalline cubic, truncated dodecahedra, and rhombic dodecahedra were discovered over time, as shown in Figure 3a. On the other hand, decreasing the sublimation rate ensured that fewer reactive sites were exposed for the expected reduction in molecular collisions, and as a result, a thermodynamic equilibrium state can be reached with the formation of a stable rhombic dodecahedral morphology and increased growth over time. The same hypothesis was further confirmed by the concentration parameter adjustment, and a solid solution with a low zinc ion concentration showed similar reduced molecular collisions during the reaction and established an equilibrium state in terms of thermodynamic control, resulting in the same stable crystalline morphologies as those obtained with a slow sublimation rate. The metastable morphologies of solid solutions with high zinc ion concentrations, with the anticipated results, were similar to those of solid solutions with high sublimation rates and kinetic control. On the basis of the described mechanism of the vapor sublimation rate vs crystalline morphology, as shown in Figure 3b, controlling the crystalline hierarchy of multiple scales of crystal sizes was performed for the MOF coatings on the same substrates. SEM images revealed homogeneous and localized control of the MOF coatings with administered scales of crystalline material fabricated at selected locations on the same substrate. Additional structural characteristics of the fabricated MOF coatings were analyzed by using combined X-ray diffraction (XRD), thermogravimetric analysis (TGA), and Fourier transform infrared spectroscopy (FTIR). The structures of the proposed MOF coatings were confirmed to be pure in crystallinity for the selected metal cores used for MOF coating fabrication. Furthermore, the experimental XRD patterns were compared with the simulated XRD pattern derived from the reported MOF structure, supporting the confirmation of the phase purity. These additional data are included in the Supporting Information in Figures S5.

**Figure 3:**
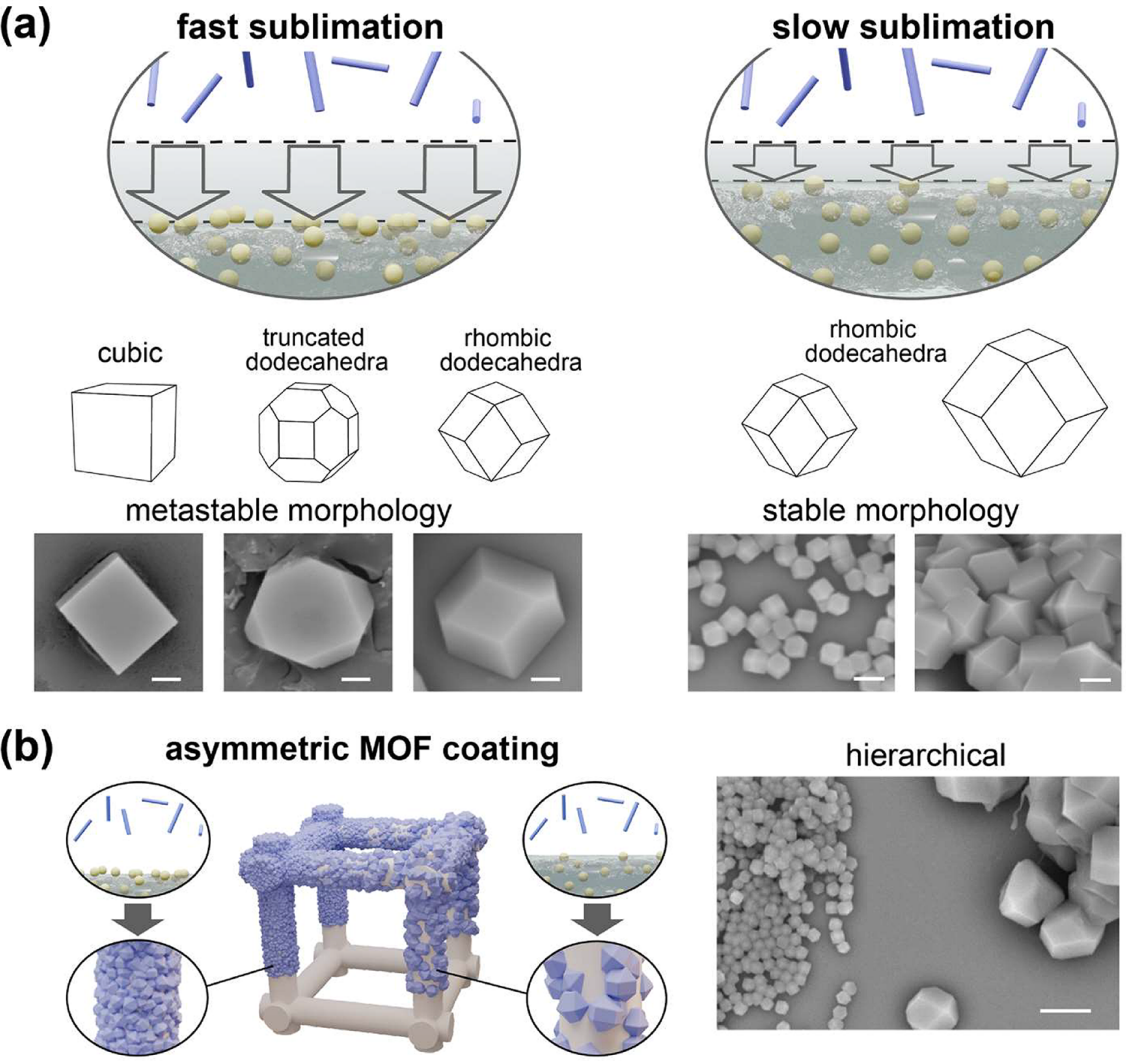
Crystalline structures of the vapor sublimation rate. (a) Schematics and SEM images showing the crystalline morphologies of the MOF coatings, which were governed by a mechanism influenced by the sublimation rate during the fabrication process. (b) Controlling the crystalline hierarchy of multiple scales of crystal sizes was performed for the MOF coatings on the same substrates. SEM images revealed homogeneous and localized control of the MOF coatings with administered scales of crystalline material fabricated at selected locations on the same substrate. Scale bars are (a) 2 and (b) 5 μm.

On the basis of the mechanism described above and the reaction dynamics of the proposed vapor-phase process, we hypothesized the accessibility of a stagewise secondary, ternary, and/or more reaction pathway, i.e., by (1) successively forming a solid solution with varying metal composition and/or vapor composition of devised linkers on the same substrate surface; (2) geographical and architectural formation of the solid solutions in compartmentalized and localized arrangements used as the sublimation counterparts; and (3) combinations of (1) and (2). The refined process enables the fabrication of functional and combinatorial MOF coatings with configured special arrangements and/or the compartmentalization of various MOF compositions. In the experiment, zinc- and cobalt-ion solutions were used to form iced solid solutions for the sublimation processes, with subsequent exposure to vapor deposition of organic linkers of 2-methylimidazole. A templated zinc solid solution was sublimated with the vapor deposition of 2-methylimidazole to form a zinc-MOF on the substrate surface (layer 1); subsequently, a second cobalt solution with the same 2-methylimidazole (theoretically replaceable with a distinct linker) was used, completing layer 2 of the cobalt-MOF on top of layer 1. A layered MOF coating configuration with a theoretically desired number of layers and compositions can be achieved by repeating the process described above in (1) and (2), as shown in Figure 4a. The resulting zinc-MOF and cobalt-MOF layered structures are also shown, with cross-sectional EDS mapping confirming the layered distribution on the basis of the elemental signals of zinc and cobalt. Similarly, a more complicated arrangement and compartmentalization of zinc and cobalt solutions and thus zigzag hierarchical MOF coatings composed of localized zinc and cobalt-MOF compartments within the overall coating layer were constructed, as shown in Figure 4b. An interesting experiment was also performed by mixing zinc and cobalt solutions for sublimation, and the proposed secondary vapor deposition process was able to construct, instead of localized compartments, an architectural bimetallic MOF coating with controlled microstructures of cubes and discs on top of the first layer, as indicated in Figure 4c. A similar secondary sublimation and deposition process was used to construct microstructured layer 2, which was composed of 300 × 300 μm cube arrays or 500 μm ⌀ disc arrays on top of homogeneous layer 1 for the proposed MOF coatings. The corresponding EDS elemental mapping for each structure is provided in the Supporting Information in Figure S6. Notably, the zinc and cobalt in the mixture both reacted with the 2-methylimidazole linker, and the resulting solid–vapor reaction is the production of bimetallic organic frameworks with theoretically varying ratios of asymmetric core metals.^55^ Moreover, they frame with two functional linkers^56^ to form one final hybrid MOF product in one reaction process. As indicated in Figure 4d, although XRD analysis confirmed that both the zinc/cobalt bimetallic MOF and the zinc- and cobalt-based monometallic MOFs exhibit similar crystalline structures, the TGA results (Figure 4e) indicate that the bimetallic MOF remains stable up to approximately 600 °C, which is attributed to stronger coordination bonds of synergistic interactions between zinc and cobalt,^57^ compared with the separate and layered zinc and cobalt-MOF coating samples that were observed with two-stage decomposition at approximately 450 °C and complete decomposition at 550 °C. The absence of distinct decomposition peaks corresponding to the individual zinc-MOF and cobalt-MOF components further supports the notion of an integrated bimetallic MOF structure rather than a physical mixture. This enhanced thermal stability highlights the potential of such bimetallic MOFs for applications requiring robust high-temperature performance.

**Figure 4:**
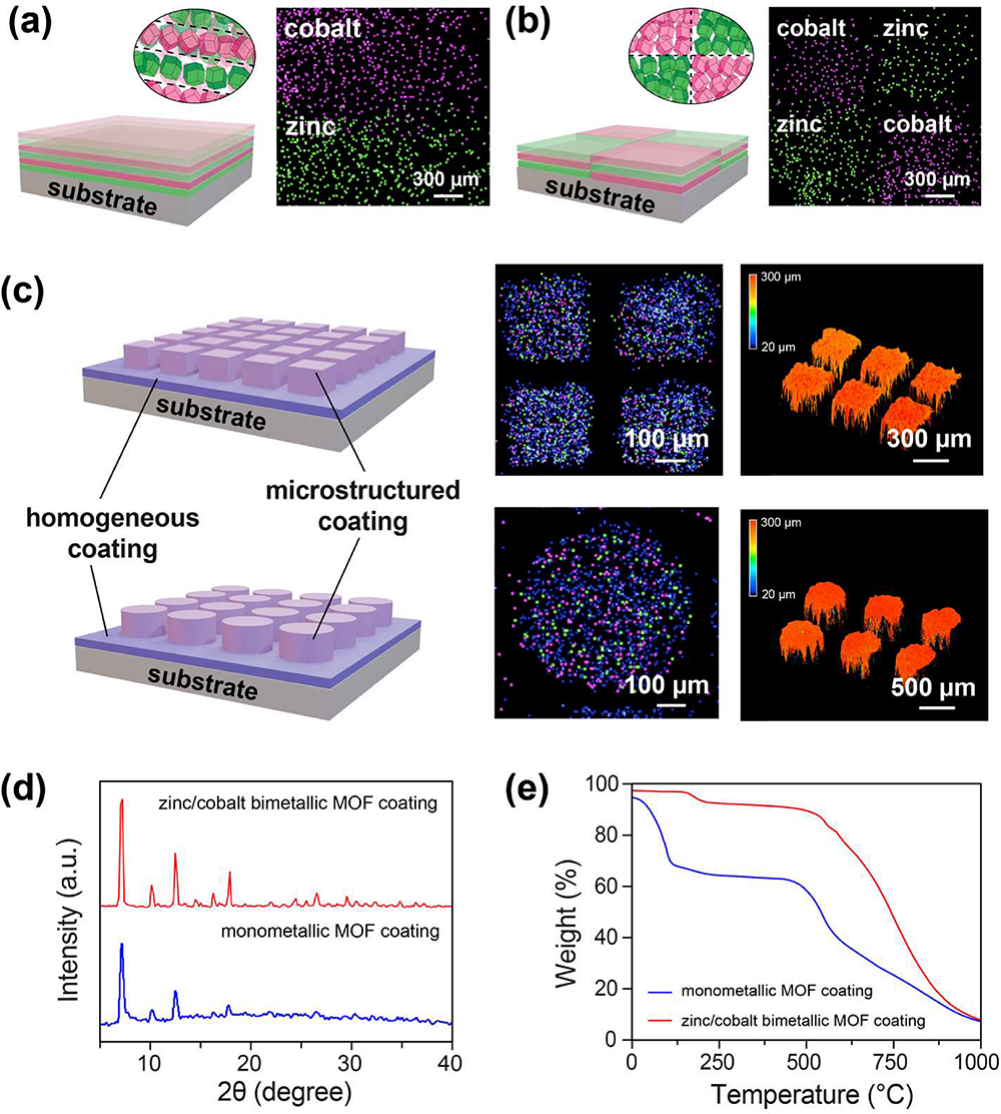
Stagewise solid–vapor reaction and the compartmentalization of MOF coatings. (a) Schematic showing the fabrication of a layered MOF coating and its configuration. Cross-sectional elemental mapping by EDS was performed to confirm that a layered architecture MOF coating was fabricated. (b) Second example of a more complicated and zigzag configuration of an MOF coating. Another EDS cross-sectional elemental mapping method also confirmed the complications of the coating layout. (c) Structured bimetallic zinc–cobalt-MOF coating fabrication with constructions of 300 × 300 μm cube arrays and 500 μm ⌀ disc arrays built on top of a homogeneous layer of a monometallic cobalt-MOF. (d) XRD analysis results for the fabricated bimetallic and monometallic MOF coatings. (e) TGA analysis revealed that the bimetallic MOF decomposes at approximately 600 °C, whereas its monometallic counterparts decompose at 450 and 550 °C.

In terms of applications, MOFs have recently attracted increasing attention in more stringent and challenging biotechnological fields because of their high efficacy in encapsulating bioactive molecules for delivery therapy, imaging diagnosis,^58^ and exterior protection of biomolecules.^59,60^ To date, the proposed vapor-phase MOF coatings are easy to fabricate on complex substrates and devices, and the coatings are conformal while keeping the process free of solvent and dry. As shown in Figure 5a, the MOF coatings were applied to substrates of 2D flat cell culture plates, 3D curved bone screws (Figure 5b), and more complicated drug-eluting stent devices (Figure 5c). Analysis and observations via microscopy indicated that the MOF coatings were homogeneous and covered the sample surfaces and were conformal coatings without compromising the topology and geometry of the substrate surfaces. The adhesion performance was further evaluated using a cross-cut tape adhesion test on the surface of a Ti_6_Al_4_V sample, as shown in Figure 5d. According to the American Society for Testing and Materials (ASTM) D3359 inspection procedure,^61^ the MOF coating exhibited no peeling from the substrate surface, demonstrating excellent interfacial adhesion to the highest inspection class of 5B in ASTM D3359. The strong adhesion of the MOF coating may be attributed to the permeability of the precursor and the anchoring effect formed after solidification^62^ and the conformal nature of the vapor deposition process,^63^ which results in the formation of coatings that can ensure long-term stability. Finally, these MOF coatings were evaluated for the ability of cells to adhere to the surface, proliferate, and differentiate. During the cell culture experiments, a zinc-MOF coating was fabricated on the cell culture dishes and was used, but not limitedly, as an example to demonstrate the proposed concept in the study. According to many studies in the literature, the release of zinc ions from MOFs can further promote cellular bioactivity, markedly increasing cell proliferation and osteogenic integration.^64,65^ In addition to ion release, the intrinsic surface structure and porosity of MOFs facilitate protein adsorption and cell adhesion, creating a favorable microenvironment for osteogenic differentiation.^66^ Furthermore, MOFs can modulate intracellular signaling pathways and enhance alkaline phosphatase (ALP) activity, thereby accelerating mineralization and bone matrix formation.^67^ As shown in Figure 5e, SEM analysis of the surface morphology of the MOF-coated samples after cell culture revealed that the residues of the cytoskeleton were well-known to have connected networks with the MOF structures, revealing the compatibility of cell adhesion. Microcomputed tomography (micro-CT) scans, on the other hand, show three-dimensional prospects of the cellular distribution within the coating layers, unambiguously demonstrating similar compatibility of cell adhesion and proliferation on the MOF coatings. Under in vitro conditions, these MOF coatings exhibit low cytotoxicity and notably offer excellent biocompatibility and increased proliferation-promoting effects for specific cell types. Unlike conventional liquid-phase MOF fabrication, which can introduce residual solvents and associated cytotoxic risks,^68^ the vapor-phase approach not only provides a dry and solvent-free process but also achieves a highly uniform and stable ultrathin coating with a thickness of approximately 1–2 μm on cell culture substrates, promoting increased material stability and significantly improving cellular adhesion. Human adipose-derived stem cells (hASCs) were cultured on MOF coatings to investigate their compatibility with osteogenic differentiation, and examinations by early stage ALP expression and alizarin red staining at the maturation stage of osteogenesis were employed to evaluate the induction of osteogenesis in the coating samples. As presented in Figure 5f, the ALP expression observed on Day 7 indicated that zinc ion release promoted hASC differentiation, and the ALP analysis on Day 14 revealed greater osteogenic activity in the MOF-containing group. To further confirm the enhancement of hASC differentiation by zinc ion release, calcium deposition on Day 21 was quantified to assess late-stage osteogenic differentiation. Statistical analysis of ALP and alizarin red staining signals additionally demonstrated that the MOF coating significantly enhanced osteogenic activity. These attributes underscore the extensive potential of zinc-MOF coatings for tissue engineering and biomedical applications, especially in modulating cell proliferation and differentiation.

**Figure 5:**
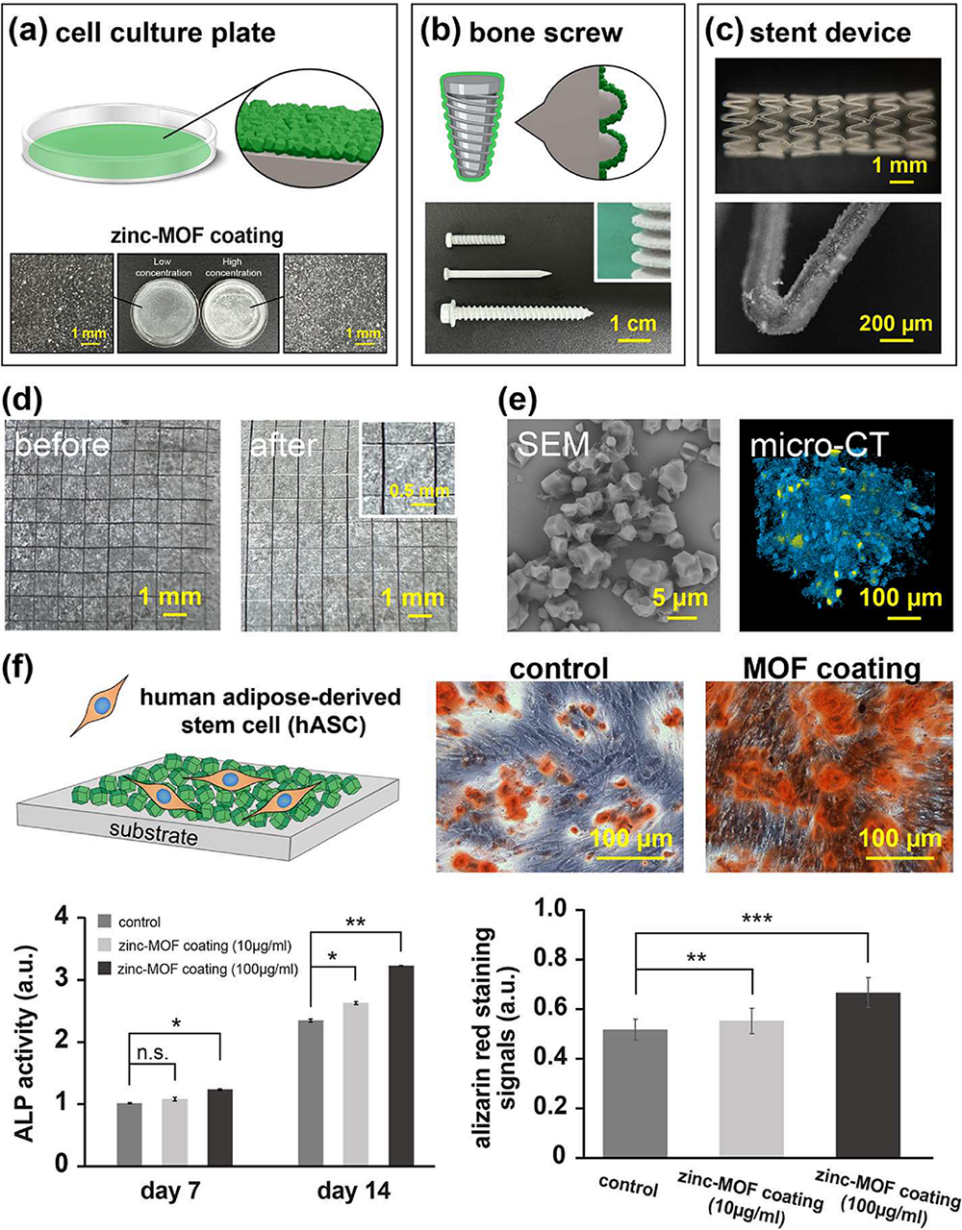
MOF coatings on 2D and 3D complex devices with durability and compatibility. Schemes and microscopy images showing the fabrication of MOF coatings on (a) cell culture plates, (b) bone screws, and (c) drug-eluting stent devices. (d) Evaluation of the mechanical stability of the MOF coatings before and after testing by using a cross-cut tape adhesion test on the Ti_6_Al_4_V substrates. (e) 2D and 3D SEM images and micro-CT scans recorded on the MOF coatings after cell culture to evaluate the compatibility of cell adhesion and differentiation. (f) Examinations of osteogenic differentiation were performed with cell cultures of adipose-derived stem cells (hASCs) on zinc-MOF coatings. Early stage osteogenesis patterns were analyzed by alkaline phosphatase (ALP) expression at Days 7 and 14 of cell culture, and mature-stage osteogenesis was evaluated by alizarin red staining on Day 21. The bar charts represent the mean values (*n* = 3) and corresponding standard deviations (±SDs) derived from three independent experiments. (n.s.: *p* value >0.05; *p value <0.05; **p value <0.01; and ***p value <0.001).

## Conclusions

The vapor-phase process has many benefits, as it is a dry process with highly controllable coating thickness and morphology, and the resulting coatings are homogeneous and have high coating fidelity toward the underlying substrate topography and geometry. Although the use of chemical vapor deposition (CVD) to prepare MOF layers has been shown to be superior to the conventional hydrothermal synthesis process, substantial knowledge and the rarity of high-cost instrument availability, which requires complex and multiple steps of processing control, and the lack of a straightforward fabrication process and direct integration with other material processing steps for wide adaptability on standard substrates and devices, these high bars in the CVD fabrication of MOFs hinder wide and practical applicability of this approach. The discovery of MOF coating fabrication from solid solutions via vapor sublimation and deposition reactions represents a general and versatile approach to generate useful MOF coatings on a variety of substrates and devices of interest. With the abundant types of MOF and linker chemistries developed already on the shelf, more prospective materials and sophisticated functional composite derivatives and rather sensitive molecules and reactions are envisioned to be produced by the proposed method. Unlimited applications using the reported technology across and combining multiple disciplines are also expected.

## Experimental Section

### Material Fabrication

#### Preparation of Zinc-MOF Coatings

A solution containing 0.075 g of zinc acetate dihydrate (Alfa Aesar, USA) was dissolved in 10 mL of deionized water and subsequently solidified with liquid nitrogen to prepare a solid solution. The frozen precursor was placed in a custom-built sublimation and deposition system, where it was exposed to 2-methylimidazole (Sigma–Aldrich, USA) serving as the linker source. To ensure a stable vapor flux during the vapor-phase reaction, 0.225 g of 2-methylimidazole was loaded in excess at the vapor inlet as the condition. Under a vacuum of 200 mTorr, the sublimation of the linker produced a stable vapor phase that facilitated the formation of zinc-MOF coatings. The vapor sublimation–deposition system consisted of a heating furnace maintained at 450 °C at the 2-methylimidazole inlet for vapor generation and a temperature-controlled sample holder kept at 0 °C to regulate the deposition process.

#### Preparation of the Cobalt-MOF Coatings

The preparation method for the cobalt-MOF coatings followed the same procedure as described for the zinc-MOF coatings, except that the precursor solution contained 0.1 g of cobalt­(II) nitrate hexahydrate (Sigma-Aldrich, USA) dissolved in 10 mL of deionized water, and 0.225 g of 2-methylimidazole was provided as the solid linker source at the vapor inlet.

### Characterizations

A real-time mass spectrometer (RGA, Hiden Analytical, UK) was used to monitor organic linker deposition under operating conditions of 10^–7^ mbar, with an electron ionization energy of 70 eV and an emission current of 20 μA. The real-time mass spectrum was reconstructed using Hiden analytic software (MASsoft7 Professional). SEM images were acquired with a Nova NanoSEM (FEI, USA) at a primary energy of 10 keV and an operating pressure of 5 × 10^–6^ Torr, while energy-dispersive X-ray spectroscopy (EDS) was performed for elemental analysis. A 300 kV FEG-TEM instrument (FEI Tecnai G2, F30) was used to capture TEM images. The material morphology was analyzed by tapping mode atomic force microscopy (Nanoscope IIIa, Veeco, Edina, MN, USA) using PointProbe Plus tips (Nanosensors, Switzerland) with a resonance frequency of 72 kHz. Crystal structure identification was conducted using a powder X-ray diffractometer (SmartLab, Rigaku, Japan) with a scanning speed of 20° min^–1^ over a 2θ range of 5°–50°. Thermogravimetric analysis was performed using an SDT 650 simultaneous thermal analyzer under a nitrogen atmosphere, which was heated from room temperature to 800 °C at a rate of 5 °C min^–1^. FTIR spectra were recorded using a Spectrum 100 FTIR Spectrometer (PerkinElmer, USA) equipped with an advanced grazing angle specular reflectance accessory (AGA, PIKE Technologies, USA). FTIR spectra were recorded with 128 scans at a resolution of 4 cm^–1^ and a wavenumber range of 500–4000 cm^–1^. Additionally, a confocal laser scanning microscope (VK-9500, Keyence, Japan) was used to construct the 3D profile of the exterior surface of the product. The 3D microstructural characteristics of the synthetic structures were analyzed using a SkyScan 2211 high-resolution micro-CT system (Bruker, Germany) with a resolution of 0.8 μm per pixel. Scanning was performed at 40 kVp and 115 μA with a 10 W output. 3D image reconstruction was carried out using GPU-Nrecon software (Bruker micro-CT, Kontich, Belgium), whereas 3D visualization was rendered using CTVox.

### Conjugations

The conjugation of fluorescent molecules, Alexa Fluor 350-labeled *N*-hydroxysuccinimide (NHS) ester and Alexa Fluor 555-labeled hydrazide (Thermo Fisher Scientific, USA), was employed to verify the presence of aminomethyl and trifluoroacetyl functionalities on cyclophane-based linkers within the MOF coatings. The reaction was carried out at 4 °C overnight. To remove excess and unreacted reagents, the conjugated samples were washed three times with phosphate-buffered saline (PBS, pH 7.4; Thermo Fisher Scientific, USA) and subsequently rinsed with deionized water. The samples were examined using a fluorescence microscope equipped with a digital camera (Leica Microsystems, Germany), and the intensities of the stained areas were analyzed using ImageJ software.

### Stability Test of the MOF Coatings

#### Adhesion Test

The stability of the MOF coatings was assessed using a multiblade cross-cut tester (ZCC 2087, Zehntner, Switzerland) on a Ti6Al4 V substrate. Two intersecting scratch patterns were made on the coated surface, followed by the application and steady removal of Scotch tape within 0.5–1.0 s at a 60° pulling angle. Adhesion performance was evaluated on the basis of the ASTM D3359 cross-cut rating scale.

#### Young’s Modulus Measurement

Young’s modulus (*E*) of the MOF coatings was determined from stress–strain curves obtained using (ElectroForce, USA). Stress (σ) was calculated by dividing the applied load by the original cross-sectional area of the sample, and strain (ε) was calculated as the ratio of change in length to the original gauge length. The initial linear elastic region of the stress–strain curve was identified visually and fitted using linear regression. Young’s modulus was calculated as the slope of this linear region:

$$E=\frac{\Delta \sigma }{\Delta \varepsilon }$$

The curve was shifted to start at (0,0) for clarity. No fracture point was observed under the chosen testing conditions.

### Cell Culture

Human adipose-derived stem cells (hASCs) were cultured in growth medium composed of Dulbecco’s modified Eagle’s medium (DMEM, HyClone, USA) supplemented with 10% fetal bovine serum (FBS, Biological Industries, Israel) and 1% antibiotic-antimycotic solution (Biological Industries, Israel). The cell suspensions were seeded onto MOF coatings at concentrations of 10 and 100 μg/mL, as well as onto tissue culture polystyrene (TCPS, Corning, USA), at a density of 1 × 10^5^ cells/cm^2^. The cultures were incubated at 37 °C in a humidified environment with 5% CO_2_ and 95% air, and the medium was changed every 3 days.

### Osteogenic

The osteogenic differentiation of hASCs was evaluated by culturing the cells in osteogenic differentiation medium. Early stage osteogenic activity was assessed by measuring alkaline phosphatase (ALP) expression using the alkaline phosphatase yellow liquid substrate system (pNPP, Sigma-Aldrich, USA) on days 7 and 14. Late-stage osteogenesis, indicated by calcium deposition, was confirmed through staining with a 1% alizarin red S solution (ARS, Sigma–Aldrich, USA) on day 21. The ALP and alizarin red staining signals were quantitatively analyzed using a microplate reader (BioTek Instruments, USA) at an absorbance wavelength of 405 nm following the manufacturer’s protocol.

### Statistical Analysis

Statistical analysis was performed using an unpaired *t* test in Prism Software version 7 (GraphPad, USA) to evaluate differences between data sets, with a significance threshold of *p* < 0.05. The data are presented as the means ± standard deviations (SDs), with each assay conducted on at least three independent samples.

## Supplementary Material



## Data Availability

All the data generated or analyzed during this study are included in this published article and its Supporting Information files.

## Notes

Residual gas analyzer characterization of functional linkers; SEM characterization of incomplete coordination of MOF coating formations; verification of functional groups on the MOF coatings; structural characterization and validation of MOF coatings; EDS elemental mapping of patterned MOF architectures; and theoretical thermodynamics and chemical reaction analysis of the mechanisms of the vapor sublimation and deposition processes (PDF)
