# Supplementary material for: Multifaced Generation of MOF Coatings via Vapor-Phase Sublimation and Deposition Reactions
Source: ACS Appl Mater Interfaces. 2025 Dec 15;18(2):4378–88. doi: 10.1021/acsami.5c16493 (PMC12828722; doi:10.1021/acsami.5c16493)
Supplement: Supplementary file 1 [file am5c16493_si_001.pdf]

Supporting Information

Multifaced Generation of MOF Coatings via Vapor-Phase Sublimation and Deposition Reactions

Shu-Man Hu[1,†], Chin-Yun Lee[1,†], Yu-Ming Chang[1], Fang-Yu Chou[2], Hui-Hsuan Wang[1], Yu-Chih Chiang[3,5,6,*], Chen-Chi Wu[4,5,*], Hsien-Yeh Chen[1,5,*]

[1] Department of Chemical Engineering, National Taiwan University, Taipei 10617, Taiwan

[2] Department of Chemical Engineering, National Taiwan University of Science and Technology, Taipei 10607, Taiwan

[3] School of Dentistry, Graduate Institute of Clinical Dentistry, National Taiwan University, Taipei 10048, Taiwan

[4] Department of Otolaryngology, National Taiwan University Hospital, Taipei 10018, Taiwan

[5] Molecular Imaging Center, National Taiwan University, Taipei 10617, Taiwan

[6] School of Dentistry, Kaohsiung Medical University, Kaohsiung 80708, Taiwan

†Shu-Man Hu and Chin-Yun Lee contributed equally to the current work.
* Corresponding Author: munichiang@ntu.edu.tw (Y.-C.C.); jimchenchiwu@gmail.com (C.-C. Wu); hsychen@ntu.edu.tw (H.-Y.C.)

KEYWORDS: metal–organic framework, coating, vapor sublimation, multifunctionality, chemical vapor deposition

Vapor sublimation rate and concentration in solid solutions

In the proposed vapor sublimation and deposition fabrication process, the sublimation of water molecules in the solid solution transitioned directly from the solid phase io the vapor phase. Under constant pressure conditions, the rate of volume change of the solid solution is controlled by the sublimation temperature and can be described by Equation (S1):

$$h(T_o - T_s) = \Delta H \rho \frac{dV}{dt} \tag{S1}$$

where T_o is the system temperature maintained at a constant value, T_s is the sublimation surface temperature, ΔH is the enthalpy of sublimation, ρ is the vapor-phase density, and dV/dt represents the rate of volume change of the solid solution.

Owing to the ongoing vapor sublimation of the solid solution, a shrinking volume and thus a proportional decrease in the surface area of the solid solution results in an increase in the metal ion concentration within the solid solution, which is also a time-dependent term, $dC(t)/dt$. With respect to the rule of mass conservation, the rate of change in the metal ion concentration can be described via Equation (S2):

$$\frac{dC(t)}{dt} = -R_s \cdot S \tag{S2}$$

where S is the surface area of the solid solution and R_s is the sublimation rate.

Partial pressure of vapor-phase linkers

During the proposed vapor sublimation and deposition fabrication process, the vapor deposition linker (e.g., 2-methylimidazole and terephthalic acid) also contributes to the balance of overall system mass conservation, and the partial pressure of these vapor phase inkers, according to Raoult's Law, can be expressed as Equation (S3):

$$P_{linker} = x_{linker} \cdot P^*_{linker} \tag{S3}$$

where P_{linker} is the partial pressure of the linker in the vapor phase, x_{linker} is the mole fraction of the linker in the solid solution, and P^*_{linker} is the saturated vapor pressure of the pure linker.

Metal ion diffusion and the MOF nucleation reaction

The diffusion of metal ions on a solid solution surface is described by Fick's Law in Equation (S4):

$$J = -D \frac{dC}{dx} \tag{S4}$$

where J represents the diffusion flux, D is the diffusion coefficient, and dC/dx denotes the

concentration gradient of metal ions in any corresponding direction.

During the vapor sublimation process, an increase in the concentration of metal ions at the solid-solution interface drives the MOF nucleation reaction. According to **classical nucleation theory**, the nucleation rate J is related to the nucleation energy barrier ΔG and is described in Equation (S5):

$$J = J_0 \exp(-\frac{\Delta G}{k_B T}) \tag{S5}$$

where J_0 is the preexponential nucleation rate constant, k_B is the Boltzmann constant, and T is the system temperature. As the concentration of metal ions increases, the nucleation energy barrier ΔG decreases, resulting in an increase in the nucleation reaction rate. Under high-concentration conditions, the nucleation process becomes kinetically controlled, resulting in rapid crystal growth and potentially irregular morphologies of the resulting MOF structures. The critical nucleation radius r^* is expressed as Equation (S6):

$$r^* = \frac{2\gamma}{\Delta G} \tag{S6}$$

A higher concentration of metal ions results in a smaller r^*, promoting finer MOF crystalline structures and ultimately influencing the pore structure and coating morphology. In contrast, under low-concentration conditions, nucleation is thermodynamically controlled, resulting in the formation of more ordered crystal structures and uniform morphologies of the MOF coatings.

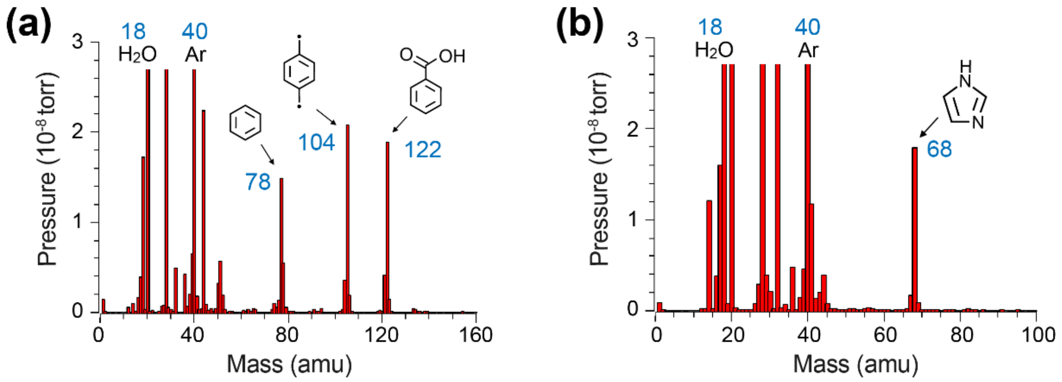

Figure S1. Verification of vapor-phase linkers by an in situ mass spectrometry gas analyzer. (a) Vaporized terephthalic acids revealed characteristic fragment peaks corresponding to benzene at 78 amu, p-xylene at 104 amu, and benzoic acid at 122 amu. (b) Vapor phase imidazole showed a characteristic peak at 68 amu.

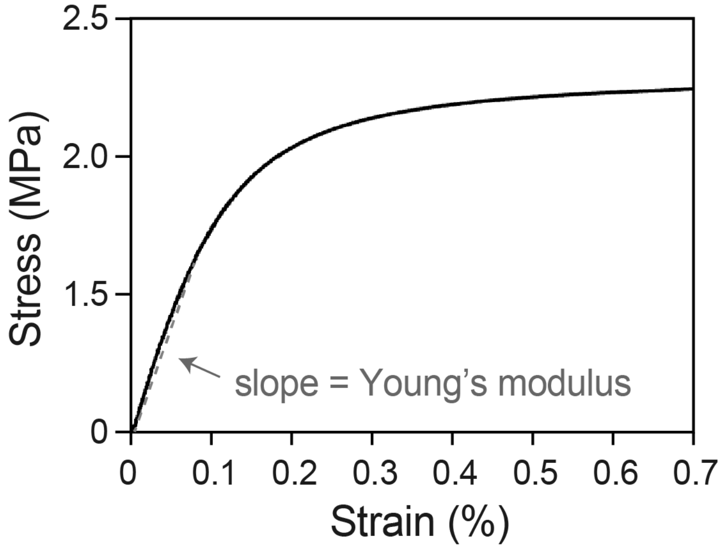

Figure S2. Stress–strain curve of zinc-MOF coatings. Stress is plotted in MPa and strain in %. Young's modulus was determined from the slope of the initial linear elastic region, as described in the experimental section. The linear fitting range is indicated by the dashed line.

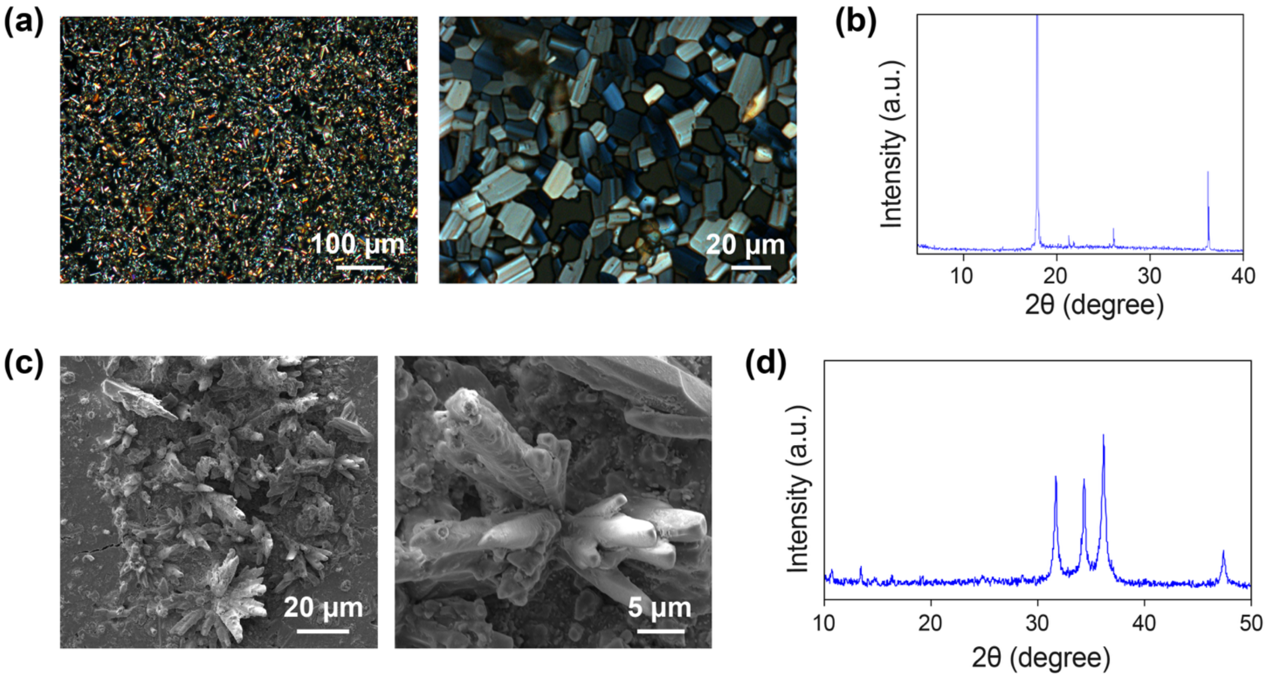

Figure S3. MOF coating fabrication under suboptimal stoichiometric conditions. (a) Polarized microscopy images reveal that crystalline precipitates from excessive and unreacted 2-methylimidazole linkers formed on the substrate surfaces. (b) Characteristic peaks were detected by XRD for the unreacted 2-methylimidazole from the samples obtained in (a). Conditions with excessive metal ions in the solid solution resulted in irregular and aggregated surface morphologies, as shown in the SEM images in (c). Uncoordinated metal ions resulted in the formation of side reactions of the ZnO byproducts that were detected by XRD in (d) with their characteristic peaks.

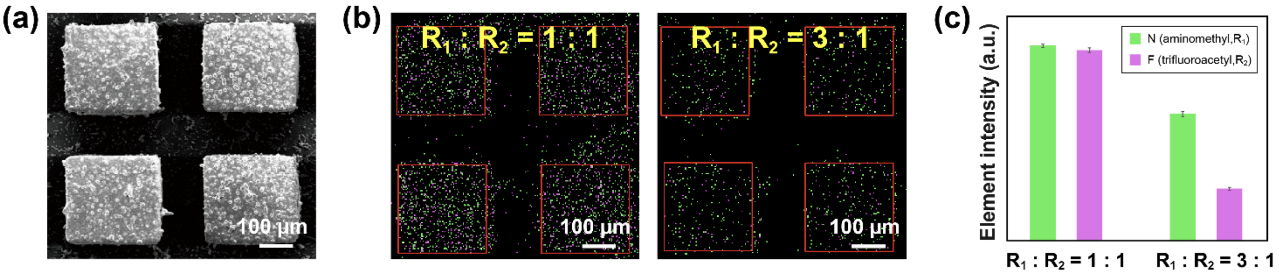

Figure S4. Additional data from SEM and EDS elemental analysis were used to confirm the composition ratios of the aminomethyl (R_1) and trifluoroacetyl (R_2) functionalities for the fabricated multifunctional MOF coatings. (a) SEM image showing the surface morphology and construction of 300x300 µm cube arrays for the coatings. The structures were generated for easy identification via EDS analysis. (b) EDS elemental mapping confirms the localized signals and intensities of nitrogen and fluorine corresponding to the R_1 and R_2 functionalities. (c) Statistical analysis of the recorded elemental intensities of nitrogen and fluorine and confirmation of the analyzed ratios of 1:1 and 3:1.

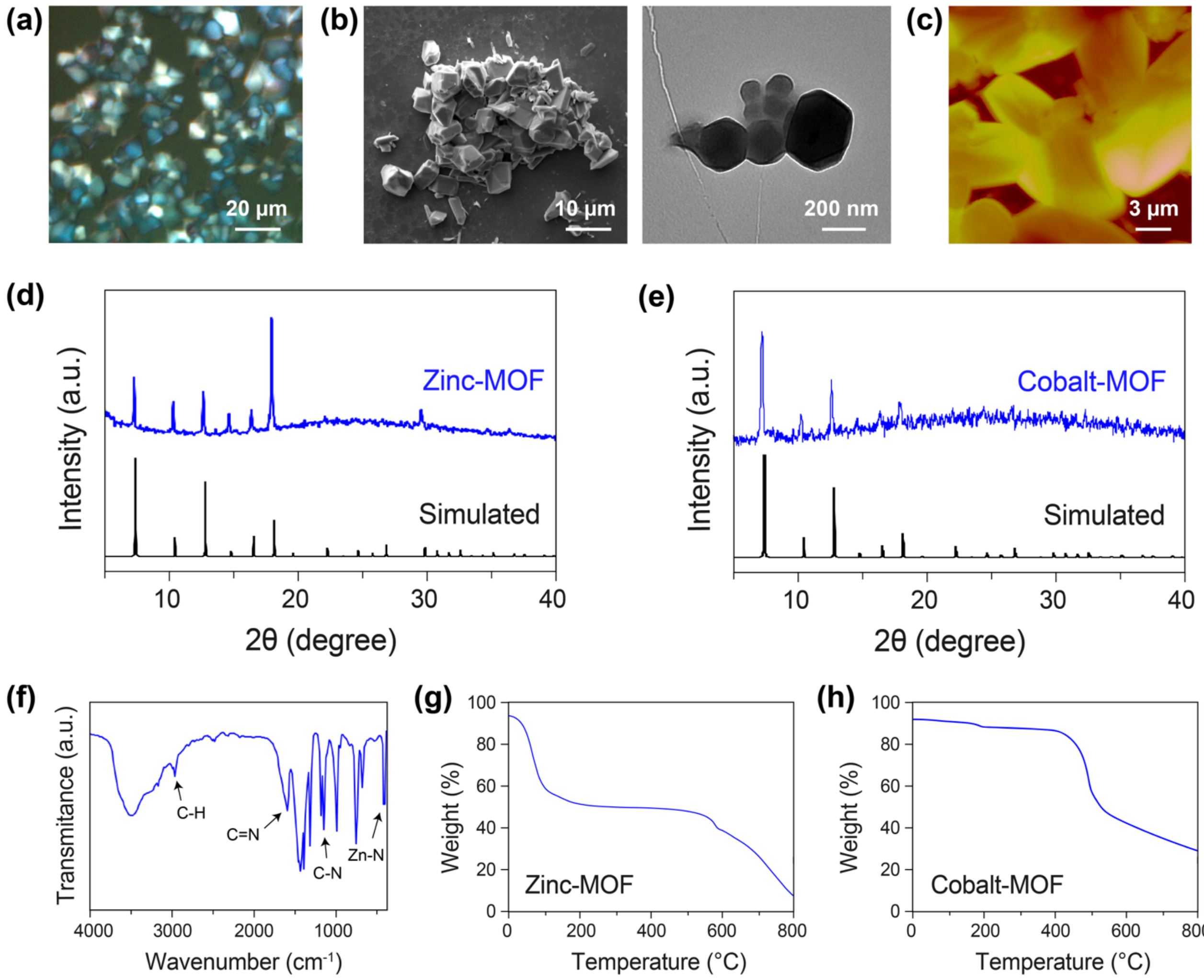

Figure S5. Morphology and structural characterization data of the MOF coatings. (a) An image obtained by polarized microscopy showing the uniform and sharp crystalline morphology of the cobalt-MOF coatings. (b) SEM and TEM images confirmed the highly crystalline morphology and structures of the zinc-MOF coatings. (c) AFM height and topography analysis revealed a uniform surface roughness and morphology profile of the zinc-MOF coatings, and a root-mean-square roughness (R_{RMS}) of 311.3 ± 20.5 nm was measured. (d) XRD patterns of the zinc-MOF coatings and the simulated pattern (CCDC- 864310) showing consistent diffraction features. (e) XRD patterns of the cobalt-MOF coatings and the simulated pattern (CCDC-671073) exhibiting matching characteristic peaks. (f) FT-IR spectra of the zinc-MOF coatings revealed characteristic vibrational peaks at 1145 cm^{-1}, 1589 cm^{-1} and 2938 cm^{-1} corresponding to C–N, C=N and C–H stretching, respectively, and a peak at 424 cm^{-1} assigned to Zn–N vibrations, confirming the formation of coordination bonds between zinc ions and

2-methylimidazole. (g) TGA curve recorded for the zinc-MOF coatings showing an initial weight loss of approximately 40% at approximately 150 °C, attributed to the evaporation of adsorbed excessive H_2O molecules. A thermal plateau up to 550 °C indicated high thermal stability. (h) TGA curves were recorded for the cobalt-MOF coatings, which indicated that the cobalt-MOF coatings were thermally stable up to approximately 450 °C.

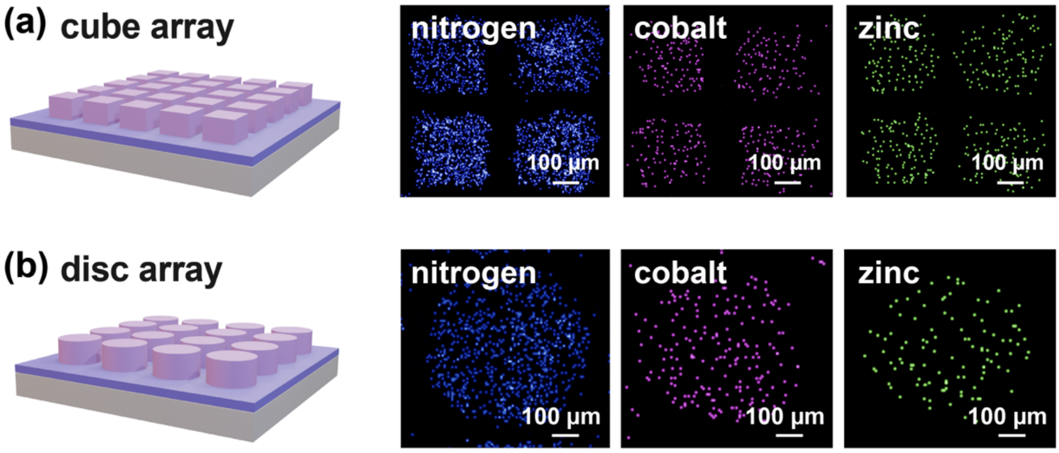

Figure S6. Additional EDS elemental analysis revealed localized nitrogen, cobalt, and zinc signals, confirming the presence of microstructured and bimetallic MOF coatings. The distributions of these element signals were consistent with the advised microstructures in (a) 300x300 µm cube arrays and (b) 500 µm⌀ disc arrays.
